# The everolimus eluting Synergy Megatron^TM^ drug‐eluting stent platform: Early outcomes from the European Synergy Megatron^TM^ Implanters' Registry

**DOI:** 10.1002/ccd.30902

**Published:** 2023-11-10

**Authors:** Kalpa De Silva, Matthew E. Li Kam Wa, Tim Wells, Abdul Mozid, Andrew Ladwiniec, Brian G. Hynes, Ashish Kotecha, Karim Ratib, Sinjini Biswas, Nicolas Amabile, Pierre Deharo, Margaret McEntagart, James C. Spratt, Franck Digne, Meadhbh Hogg, Jonathan A. Mailey, Simon J. Walsh, Sundeep S. Kalra

**Affiliations:** ^1^ Cardiovascular Division, St Thomas' Hospital Guy's and St Thomas' NHS Foundation Trust London UK; ^2^ Coronary Research Group, British Heart Foundation Centre of Research Excellence King's College London London UK; ^3^ Cardiology Department, Salisbury District Hospital Salisbury NHS Foundation Trust Salisbury UK; ^4^ Cardio Respiratory Clinical Services Unit, Leeds General Infirmary The Leeds Teaching Hospitals NHS Trust Leeds UK; ^5^ Department of Cardiology, Glenfield Hospital University Hospitals of Leicester NHS Trust Leicester UK; ^6^ Cardiology Department University Hospital Galway Galway Ireland; ^7^ Department of Cardiology, Royal Devon and Exeter Hospital Royal Devon University Healthcare NHS Foundation Trust Exeter UK; ^8^ Cardiology Department, Royal Stoke University Hospital University Hospitals of North Midlands NHS Trust Stoke UK; ^9^ Bristol Heart Institute University Hospitals Bristol NHS Foundation Trust Bristol UK; ^10^ Cardiology Department L'Institut Mutualiste Montsouris Paris France; ^11^ Cardiology Department Assistance Publique Hôpitaux de Marseille Marseille France; ^12^ Cardiology Department, Golden Jubilee Hospital NHS Golden Jubilee Glasgow UK; ^13^ Cardiology Clinical Academic Group, St George's University Hospital St George's University Hospitals NHS Foundation Trust London UK; ^14^ Cardiology Department Centre Cardiologique du Nord Saint Denis France; ^15^ Department of Cardiology Belfast Health and Social Care Trust Belfast UK; ^16^ Cardiology Department, Royal Free Hospital Royal Free London NHS Foundation Trust London UK

**Keywords:** bifurcation, drug‐eluting stent, left main

## Abstract

**Background:**

The Synergy Megatron^TM^ is an everolimus‐drug eluting stent that may offer advantages in the treatment of aorto‐ostial disease and large proximal vessels.

**Aims:**

To report the short‐ to medium‐term clinical outcomes from the European Synergy Megatron^TM^ Implanters' Registry.

**Methods:**

This registry was an investigator‐initiated study conducted at 14 European centers. The primary outcome was target lesion failure (TLF), defined as the composite of cardiovascular death, target vessel myocardial infarction (MI), and target lesion revascularisation.

**Results:**

Five hundred seventy‐five patients underwent PCI with Megatron^TM^ between 2019 and 2021. Patients were 69 ± 12 years old, 26% had diabetes mellitus, 24% had moderate‐severe left ventricular impairment and 59% presented with an acute coronary syndrome. 15% were deemed prohibitively high risk for surgical revascularisation. The target vessel involved the left main stem in 55%, the ostium of the RCA in 13% and was a true bifurcation (Medina 1,1,1) in 50%.  At 1 year, TLF was observed in 40 patients, with 26 (65%) occurring within the first 30 days. The cumulative incidence of TLF was 4.5% at 30 days and 8.6% (95% CI 6.3–11.7) at 1 year. The incidence of stent thrombosis was 0.5% with no late stent thromboses. By multivariate analysis, the strongest independent predictors of TLF were severe left ventricular impairment (HR 3.43, 95% CI: 1.67–6.76, *p* < 0.001) and a target vessel involving the left main (HR 4.00 95% CI 1.81–10.15 *p* = 0.001).

**Conclusions:**

Use of the Synergy Megatron^TM^ everolimus eluting stent in a ‘real‐world’ setting shows favorable outcomes at 30 days and 1 year.

**Abbreviations**
ACS: Acute coronary syndrome
LM: Left main
LSD: Longitudinal stent deformation
LV: Left ventricular
PCI: Percutaneous coronary intervention
STEMI: ST‐elevation myocardial infarction
TLF: Target lesion failure

## 1 INTRODUCTION

Percutaneous coronary intervention (PCI) is undertaken in increasingly high‐risk patients and complex coronary disease, including aorto‐ostial, left main (LM) and bifurcation disease.^1^, ^2^ There are fundamental differences in the arterial structure and composition of these lesions, versus those that are located in the rest of the coronary tree. A predominance of fibrotic and calcified tissue reduces arterial compliance^3^ and resists balloon dilatation, with chronic recoil contributing to increased rates of restenosis.^4^ Combined with the challenges of vessel eccentricity and angulation, PCI to these lesions is associated with inferior long term clinical outcomes.^3^, ^5^, ^6^, ^7^

The Synergy Megatron^TM^ is an evolution of the Synergy abluminal coated, biosorbable polymer, everolimus‐eluting, platinum chromium stent. Modifications versus the Synergy Large Vessel (4.0–5.0 mm nominal diameter) include more peaks (12 vs. 10), more connectors in the stent body (3 vs. 2), and increased strut thickness (89 vs. 81 µm) for a manufacturer reported increase in axial and radial strength on bench testing. A single platform allows overexpansion from 3.5 to 6.0 mm, such as when there is significant mismatch between the LM and its daughter vessels.^8^

However, whilst these features are theoretically attractive, the outcomes of the Megatron^TM^ stent remain to be described outside of case reports and small studies.^9^, ^10^, ^11^ To address this, we report the early‐ to medium‐term clinical and procedural outcomes from unselected, real‐world patients, enrolled in a multicentre European registry.

## 2 METHODS

### 2.1 Study design and patient population

The European Synergy Megatron^TM^ Implanters' Registry is an investigator initiated, retrospective, international, multicentre registry conducted at 14 centers in France, Ireland and the United Kingdom. Consecutive patients undergoing clinically indicated PCI between September 2019 and July 2021 with the Megatron^TM^ stent were included in the registry. All patients received dual antiplatelet therapy for the duration of follow up in line with current ESC guidelines.^12^ The device manufacturer, Boston Scientific, had no role in study design, data analysis or control over manuscript publication.

Due to the retrospective nature of the study, patients were not approached for consent. All sites complied with local institutional review board and relevant national ethical requirements for the processing of fully anonymised data, which was transferred to King's College London, UK for final analysis.

### 2.2 Outcomes and definitions

The primary outcome for this study was target lesion failure (TLF), defined as cardiovascular death, target vessel myocardial infarction and target vessel revascularisation. The secondary outcome was patient‐orientated composite events (POCE), defined as a composite of all‐cause mortality, stroke, any myocardial infarction, and any revascularisation.

These endpoints, as well as target lesion revascularisation and stent thrombosis are as defined in the Academic Research Consortium (ARC)‐2 consensus document.^13^ Procedural success was defined as the implantation of the Megatron^TM^ stent without any complications that required additional percutaneous or surgical intervention.

Use of the Megatron^TM^ was at the discretion of the operator. Given its indication for large proximal vessels, we stratified outcomes according to target lesion characteristics. Complex lesions were defined as those involving the left main, the ostium of the right coronary artery, or bifurcations. The remaining lesions were defined as noncomplex.

### 2.3 Statistical analysis

Continuous variables are presented as mean ± standard deviation and compared using the Student's unpaired *t*‐test. Categorical variables are presented as counts and percentages (of available data when incomplete). They were compared using the Mann‐Whitney, or Fisher's exact tests as appropriate. Cumulative event rates were calculated using the Kaplan‐Meier method and comparisons made between groups using the log‐rank test. Predictors of TLF were identified by Cox proportional hazards analysis. Covariates were first screened in univariate models and those that were both significant (*p*‐value < 0.10) and judged to be clinically important were included in a multivariate analysis. This was used to estimate hazard ratios (HR) and 95% confidence intervals (CI). A probability value of <0.05 was considered significant. All data were analyzed using GraphPad Prism 9.5.1 (GraphPad Software, CA, USA).

## 3 RESULTS

Five hundred seventy‐five patients underwent PCI with Megatron^TM^ during the study period. Clinical outcomes were available at a median of 365 days (IQR 30‐365) with 30‐day and 1‐year follow up available in 100% and 58%, respectively. Baseline characteristics are detailed in Table 1 and stratified by target lesion complexity in Table S1. The mean age was 68.5 ± 11.5 years and 83% were male. Sixty‐two percent were hypertensive, 26% were diabetic and 41% had a history of previous percutaneous or surgical revascularisation. 42% had left ventricular impairment and 59% presented with an acute coronary syndrome (ACS). Of those presenting with ACS, 34% were STEMI and 57% involved intervention to the LM.

**Table 1:** Baseline patient characteristics.

	*N* = 575
Age (years)	68.5 ± 11.5
Male	456 (83)
Hypertension	341 (62)
Dyslipidaemia	328 (60)
Diabetes	142 (26)
End Stage Renal Failure	19 (3)
Previous MI	139 (25)
Previous PCI	174 (32)
Previous CABG	50 (9)
Previous CVA	57 (10)
PVD	55 (10)
BMI	28.0 ± 6.9
LV function	
Normal	313 (58)
Mild‐Moderate impairment	173 (32)
Severe impairment	54 (10)
Clinical Syndrome	
Stable angina	223 (41)
ACS ‐ NSTEMI/UA	215 (39)
ACS ‐ STEMI	112 (20)

Abbreviations: BMI, body mass index; CABG, coronary artery bypass graft; CVA, cerebrovascular accident; MI, myocardial infarction; NSTEMI, non‐ST elevation myocardial infarction; PCI, percutaneous coronary intervention; PVD, peripheral vascular disease; STEMI, ST elevation myocardial infarction.

Procedural characteristics are described in Table 2. The target vessel for the Megatron^TM^ stent involved the LM in 55% and the ostium of the RCA in 13%. Fifty percent of targets were bifurcation lesions (93% of these LM bifurcation). Intravascular imaging was used in 78% of all LM cases, with an overall rate of 65% within the whole cohort. Procedural success was achieved in 98%.

**Table 2:** Procedural characteristics.

	*N* = 575
Surgical turndown	82 (15)
Radial access	496 (91)
Target vessel	
Isolated left main	27 (5)
Left main and LAD/LCx	290 (50)
Ostial RCA	75 (13)
Saphenous vein graft	6 (1)
Chronic total occlusion	45 (8)
Bifurcation	286 (50)
Provisional	202 (35)
Upfront 2 stent	84 (15)
For in‐stent restenosis	35 (6)
Number of stents	1.9 ± 1.1
Stented segment length	46.9 ± 36.0
Intravascular imaging	359 (65)
IVUS	325 (59)
OCT	34 (6)
Post dilatation	478 (87)
Procedural success	562 (98)

Abbreviations: IVUS, intravascular ultrasound; OCT, optical coherence tomography.

### 3.1 Clinical outcomes

**Figure 1:**
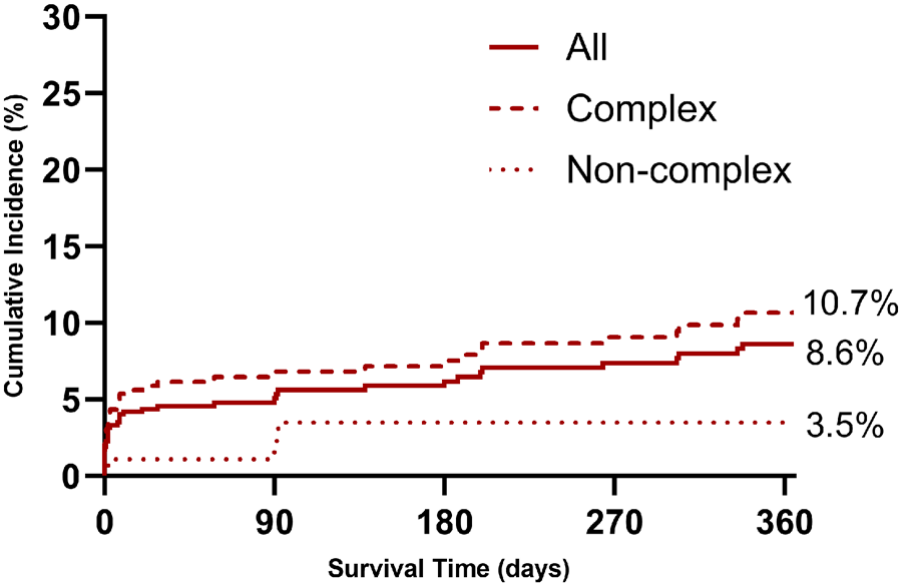
Kaplan‐Meier estimated cumulative incidence rate of TLF up to 1 year and stratified by target lesion complexity. Complex versus noncomplex Log‐rank *p* = 0.008. ACS, acute coronary syndrome; LM, left main; STEMI, ST elevation myocardial infarction. [Color figure can be viewed at wileyonlinelibrary.com]

**Figure 2:**
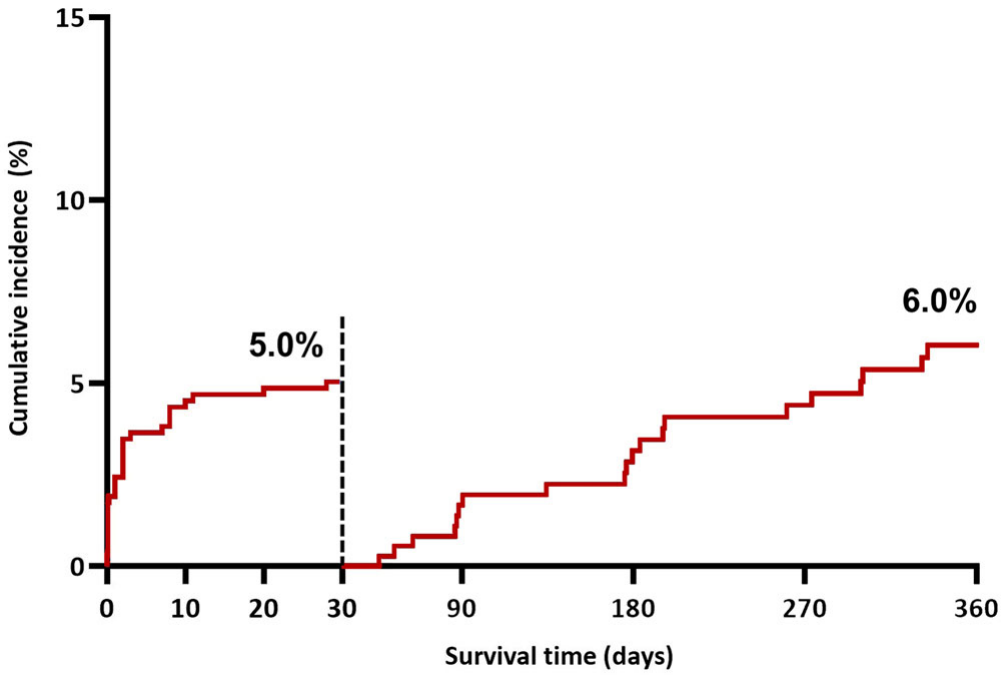
Landmark analysis of TLF at 30 days after Megatron^TM^ implantation. Cumulative incidence and Kaplan‐Meier event rates of the primary outcome (target lesion failure) within and after 30 days. [Color figure can be viewed at wileyonlinelibrary.com]

TLF occurred in 40 of 575 patients at a median of 8 (IQR 0.1–127) days after Megatron^TM^ implantation. The cumulative incidence of TLF by the Kalpan‐Meier method was 8.6% (95% CI 6.3–11.7) at 1 year. TLF was higher with complex versus noncomplex target lesions (10.7% vs. 3.5%, *p* = 0.008) (Figure 1). Landmark analysis shows that 26 of the 40 (65%) events, occurred within 30 days of the procedure, corresponding to an event rate of 4.7% (Figure 2). The cumulative incidence of POCE was 4.9% at 30 days and 10.6% (95% CI 8.0–13.4) at 1 year.

Early TLF was driven by cardiovascular death which occurred in in 17 (65%) patients. Myocardial infarction occurred in the remaining nine (35%) patients, six of which were periprocedural. The remaining target vessel myocardial infarctions included two acute, and one subacute stent thromboses. One of these occurred within a Megatron^TM^ stent. This was an acute thrombosis within a stent that had been placed across the LM‐LAD bifurcation during primary PCI for STEMI in a patient with severe LV dysfunction. After 30 days, cardiovascular death occurred in a further six patients and spontaneous target vessel myocardial infarction in five patients. Target lesion revascularisation occurred in seven patients. There were no cases of late stent thrombosis.

Patients who experienced TLF were more likely to be diabetic with a history of MI, and severe impairment of LV function. (Table 3). They were more likely to have undergone PCI that involved the left main or for in stent restenosis (Table 4).

**Table 3:** Comparison of patient characteristics between those with and without 1‐year TLF.

	TLF (*N* = 40)	No TLF (*N* = 535)	*p*‐value
Age (years)	70.8 ± 9.0	67.2 ± 11.6	0.105
Male	32 (80)	424 (83)	0.612
Hypertension	25 (63)	316 (62)	0.999
Dyslipidaemia	23 (58)	305 (60)	0.867
Diabetes	16 (40)	126 (25)	0.040*
End Stage Renal Failure	3 (8)	16 (3)	0.153
Previous MI	17 (43)	122 (24)	0.014*
Previous PCI	14 (35)	160 (31)	0.724
Previous CABG	6 (15)	44 (9)	0.245
Previous CVA	7 (18)	50 (10)	0.245
PVD	5 (13)	50 (10)	0.582
BMI	25.8 ± 8.5	28.1 ± 6.8	0.054
LV function			
Normal	17 (43)	296 (59)	0.101
Mild‐Moderate impairment	11 (28)	161 (32)	0.859
Severe impairment	12 (30)	43 (9)	<0.001*
Clinical Syndrome			
Stable angina	12 (30)	211 (41)	0.158
ACS ‐ NSTEMI/UA	15 (38)	200 (39)	0.868
ACS ‐ STEMI	13 (33)	99 (19)	0.064

*Note*: Percentages may not total 100% due to rounding and counts may be less than the total participants in cases of incomplete data.

Abbreviations: BMI, body mass index; CABG, coronary artery bypass graft; CVA, cerebrovascular accident; MI, myocardial infarction; NSTEMI, non‐ST elevation myocardial infarction; PCI, percutaneous coronary intervention; PVD, peripheral vascular disease; STEMI, ST elevation myocardial infarction.

**Table 4:** Comparison of procedural characteristics between those with and without 1‐year TLF.

	TLF (*N* = 40)	No TLF (*N* = 535)	*p*‐value
Surgical turndown	10 (26)	72 (14)	0.063
Radial access	32 (82)	464 (91)	0.082
Target vessel			
Isolated left main	2 (5)	25 (5)	0.925
Left main and LAD/LCx	33 (83)	284 (53)	<0.001*
Ostial RCA	3 (8)	72 (13)	0.462
Saphenous vein graft	1 (3)	5 (1)	0.353
Chronic total occlusion	2 (5)	43 (8)	0.762
Bifurcation			
Provisional	19 (48)	183 (34)	0.121
Upfront 2 stent	10 (25)	74 (14)	0.063
For in‐stent restenosis	6 (15)	29 (6)	0.030*
Number of stents	2 (1‐3)	2 (1‐3)	0.158
Stent length	54.1 ± 49.5	46.3 ± 34.7	0.189
Intravascular imaging			
IVUS	27 (68)	298 (58)	0.317
OCT	2 (5)	32 (6)	0.999
Post dilatation	38 (95)	439 (86)	0.145

*Note*: Percentages may not total 100% due to rounding and counts may be less than the total participants in cases of incomplete data.

Abbreviations: IVUS, intravascular ultrasound; OCT, optical coherence tomography.

After multivariate analysis, previous MI, severe LV impairment, STEMI, target vessel involving the left main stem, and PCI for in stent restenosis were independently associated with an increased risk of TLF at 1 year (Table 5).

**Table 5:** Univariate and multivariate Cox proportional hazards analysis of 1‐year TLF.

	Univariate		Multivariate	
	HR (95% CI)	*p*‐value	HR (95% CI)	*p*‐value
Diabetes	1.94 (1.01–3.62)	0.040	1.54 (0.78–2.92)	0.195
Previous MI	2.23 (1.17–4.15)	0.012	2.34 (1.17–4.61)	0.015*
Severe LV impairment	4.02 (1.97–7.72)	<0.001	3.45 (1.67–6.76)	<0.001*
STEMI	1.93 (0.97–3.67)	0.051	2.82 (1.36–5.57)	0.004*
Target involving LM^23^, ^24^	3.65 (1.71–9.00)	<0.002	4.00 (1.81–10.15)	0.001*
For ISR	2.68 (1.01–5.95)	0.026	2.72 (0.97–6.51)	0.036*

Abbreviations: CVA, cerebrovascular accident; ISR, in stent restenosis; LAD, left anterior descending; LCx, left circumflex; LM, left main; LV, left ventricular; STEMI, ST elevation myocardial infarction.

* Significant with *p* < 0.05 after multivariate analysis.

## 4 DISCUSSION

Use of the Synergy Megatron^TM^, in a complex and real‐world population is associated with acceptable short term clinical outcomes at 30 days and 1 year. To our knowledge, this is the largest study reporting on the clinical outcomes of this platform. The factors independently associated with TLF in this study are well established predictors of poor short‐term outcomes.^14^, ^15^, ^16^

Aorto‐ostial lesions present several obstacles that must be overcome during PCI. Eccentricity and the “funnel” shape of the LM, combined with the fundamental limitations of two‐dimensional angiography contribute to high rates of geographical miss in these lesions.^17^ The distal left main stem involves the largest coronary bifurcation of all, and two further ostia. These share the histological features of fibrosis and calcification as the aorto‐ostial junction, but may also be combined with severe angulation and a large mismatch in size, increasing the PCI technical complexity.^6^ Despite this, it is the ostium of the right coronary that appears to be associated with the highest rates of TLF, with stent fracture identified in half of those presenting with ISR, in one series.^18^

Contemporary thin strut drug‐eluting stents whilst highly deliverable, are used in all coronary segments without regard for these differences, however. The Synergy Megatron is designed to address these specific issues. The increased radial and axial strength of this platform may resist vessel recoil as well as longitudinal stent deformation (LSD). LM intervention significantly increases the risk of LSD,^19^ which was observed in 6.6% of final IVUS images from the EXCEL trial. This was associated with an increase in cardiac death, MI, and ischemia driven target lesion revascularisation at 3 years (28.3% vs. 13.9%, *p* = 0.02) as well as numerically greater stent thrombosis, regardless of stent area or protrusion into the aorta.^20^ Correct use of the proximal optimization technique (POT) is mandatory to appose the stent within the proximal main vessel, facilitate entry into the side branch, and to avoid disruption to the neocarina. However, the reduction of metal and eluted drug to vessel ratio from the POT has also been suggested as a potential source of long‐term device failure.^21^, ^22^ The Megatron's single platform allows over‐expansion to 6 mm, suited to adequately scaffolding large proximal vessels despite any size mismatch between the LM and the left anterior descending (LAD) or circumflex (LCx) arteries.

Given the Megatron's^TM^ indication for aorto‐ostial, left main bifurcation, and proximal disease, the results are perhaps best considered in comparison to the 30‐day and 1‐year MACE outcomes from the PCI arms of NOBLE (4% and 8%) and EXCEL (5% and 8%)^23^, ^24^; the 1 year composite primary outcome of EBC MAIN (14.7% within the stepwise provisional group)^25^; as well as a recent left main analysis of the Swedish Coronary Angiography and Angioplasty (SCAAR) registry.^1^ In common with the SCAAR registry, which reported a 14% and 24% MACE rate, with a 7.9% rate of periprocedural complications, this study included surgical turndown (15%), with a high proportion of co‐morbidities and acute coronary syndromes. Compared to these studies, the 30‐day TLF rate of 4.7%, 1 year rate of 8.6%, and periprocedural complication rate of 2.3% in this study are highly favorable. We show that the majority of events occur early, particularly within the first 10 days. This is therefore likely to reflect the underlying presentation, rather than the effect of any treatment strategy or device such as the Megatron^TM^.^26^, ^27^, ^28^, ^29^ The early stent thrombosis rate within the whole cohort was 0.5%, similar to the 0.2%–0.6% reported in other studies.^23^, ^24^, ^29^ These results should perhaps be considered unsurprising given that the Megatron^TM^ is an iteration of the Synergy platform, with an established safety record.^30^, ^31^, ^32^ Nonetheless, it is seems probable that the purported resistance to recoil and deformation of this new platform will only yield detectable clinical benefits with long term follow up.

Although these indirect comparisons with existing data are hypothesis generating, true differences in meaningful clinical outcomes can only be assessed in the context of a prospective randomized trial.

### 4.1 Limitations

This study has limitations that are inherent to all observational registries. Although consecutive patients have been enrolled at each centre, the decision to use a Megatron^TM^ was at the operator's discretion, introducing selection bias. Although we consider the reporting of all cause death to be robust, adjudication of cardiovascular versus non‐cardiovascular death was not performed by a clinical events committee. Assessment of periprocedural myocardial infarction was not mandated by protocol. Measures of procedural complexity such as the use of calcium modification devices and anatomical SYNTAX score were not available as these are not routinely collected across all national registries for the participating centers. These findings should be considered most robust for short term outcomes, given the loss to follow up at 1 year. Finally, although this is the largest published series to date, the numbers are nonetheless relatively small.

## 5 CONCLUSIONS

We report the largest real‐world registry providing evidence of the safety and clinical performance of Megatron^TM^, in a real‐world, complex patient population, with satisfactory 30‐day and 1‐year clinical outcomes. Additional prospective, randomized data is required to further assess this novel stent platform in the setting of aorto‐ostial coronary disease.

## CONFLICT OF INTEREST STATEMENT

KDS and ML are supported by the British Heart Foundation (RE/18/2/34213, FS/CRTF/22/24342). MM, JCS, and FD report honoraria, speaker, or consultancy fees from Boston Scientific. SJW is currently an employee of Boston Scientific. The remaining authors declare no conflict of interest.

## Supporting information

Supporting information.

## Data Availability

The data that support the findings of this study are available from the corresponding author upon reasonable request.

## References

[1] Mohammad MA , Persson J , Buccheri S , et al. Trends in clinical practice and outcomes after percutaneous coronary intervention of unprotected left main coronary artery. J Am Heart Assoc. 2022;11:24040. 10.1161/JAHA.121.024040 PMC9075483 35350870

[2] Vora AN , Dai D , Gurm H , et al. Temporal trends in the risk profile of patients undergoing outpatient percutaneous coronary intervention: a report from the National Cardiovascular Data Registry's CathPCI Registry. Circul Cardiovasc Interv. 2016;9(3):e003070. 10.1161/CIRCINTERVENTIONS.115.003070 26957417

[3] Jaffe R , Halon DA , Shiran A , Rubinshtein R . Percutaneous treatment of aorto‐ostial coronary lesions: current challenges and future directions. Int J Cardiol. 2015;186:61‐66. 25814346 10.1016/j.ijcard.2015.03.161

[4] Tsunoda T , Nakamura M , Wada M , et al. Chronic stent recoil plays an important role in restenosis of the right coronary ostium. Coron Artery Dis. 2004;15:39‐44. 15201619 10.1097/00019501-200402000-00006

[5] Hsieh IC , Chen CC , Chang SH , et al. Acute and long‐term outcomes of drug‐eluting stent implantations in aorto‐ostial, left anterior descending artery‐ostial, and nonostial lesions. Catheter Cardiovasc Interv. 2013;82:727‐734. 23592601 10.1002/ccd.24943

[6] Musallam A , Chezar‐Azerrad C , Torguson R , et al. Procedural outcomes of patients undergoing percutaneous coronary intervention for De Novo lesions in the ostial and proximal left circumflex coronary artery. Am J Cardiol. 2020;135:62‐67. 32958219 10.1016/j.amjcard.2020.08.014

[7] Hyun J , Kim JH , Jeong Y , et al. Long‐term outcomes after PCI or CABG for left main coronary artery disease according to lesion location. JACC: Cardiovasc Interv. 2020;13:2825‐2836. 33357520 10.1016/j.jcin.2020.08.021

[8] Boston Scientific. SYNERGY MEGATRON^TM^ Stent System n.d. Accessed November 17, 2022. https://www.bostonscientific.com/en-EU/products/stents-coronary/synergy-stent-system/megatron.html

[9] Samant S , Wu W , Zhao S , et al. Computational and experimental mechanical performance of a new everolimus‐eluting stent purpose‐built for left main interventions. Sci Rep. 2021;11:8728. 33888765 10.1038/s41598-021-87908-2 PMC8062511

[10] Mailey JA , Ahmed M , Hogg M , et al. Initial experiences of percutaneous coronary intervention using a new‐generation Everolimus‐Eluting Stent platform. J Invasive Cardiol. 2021;33:784. 10.25270/jic/20.00663 34609325

[11] Chatzizisis YS , Makadia J , Zhao S , et al. First‐in‐human computational preprocedural planning of left main interventions using a new Everolimus‐Eluting Stent. JACC: Case Reports. 2022;4:325‐335. 35495558 10.1016/j.jaccas.2022.02.001 PMC9040115

[12] Neumann F‐J , Sousa‐Uva M , Ahlsson A , et al. 2018 ESC/EACTS guidelines on myocardial revascularization. Eur Heart J. 2018;40:87‐165. 10.1093/eurheartj/ehy855 30615155

[13] Garcia‐Garcia HM , McFadden EP , Farb A , et al. Standardized end point definitions for coronary intervention trials: the academic research consortium‐2 consensus document. Circulation. 2018;137:2635‐2650. 29891620 10.1161/CIRCULATIONAHA.117.029289

[14] Doll JA , O'Donnell CI , Plomondon ME , Waldo SW . Contemporary clinical and coronary anatomic risk model for 30‐day mortality after percutaneous coronary intervention. Circul Cardiovasc Interv. 2021;14:E010863. 10.1161/CIRCINTERVENTIONS.121.010863 34903032

[15] McAllister KSL , Ludman PF , Hulme W , et al. A contemporary risk model for predicting 30‐day mortality following percutaneous coronary intervention in England and Wales. Int J Cardiol. 2016;210:125‐132. 26942330 10.1016/j.ijcard.2016.02.085 PMC4819905

[16] Peterson ED , Dai D , DeLong ER , et al. Contemporary mortality risk prediction for percutaneous coronary intervention. JACC. 2010;55:1923‐1932. 20430263 10.1016/j.jacc.2010.02.005 PMC3925678

[17] Rubinshtein R , Ben‐Dov N , Halon DA , Lavi I , Finkelstein A , Lewis BS . Geographic miss with aorto‐ostial coronary stent implantation: insights from high‐resolution coronary computed tomography angiography. EuroIntervention. 2015;11:301‐307. 24694540 10.4244/EIJV11I3A57

[18] Yamamoto K , Sato T , Salem H , et al. Mechanisms and treatment outcomes of ostial right coronary artery in-stent restenosis. EuroIntervention. 2023;19(5):e383‐e393. 10.4244/eij-d-23-00107 37283548 PMC10397676

[19] Rhee TM , Park KW , Lee JM , et al. Predictors and long‐term clinical outcome of longitudinal stent deformation: insights from pooled analysis of Korean multicenter drug‐eluting stent cohort. Circul Cardiovasc Interv. 2017;10:e005518. 10.1161/CIRCINTERVENTIONS.117.005518 29146671

[20] Kim S‐Y , Maehara A , Merkely B , et al. TCT‐44 frequency and impact of acute stent deformation after PCI of left main coronary artery disease: an EXCEL trial intravascular ultrasound substudy. JACC. 2017;70:B19.

[21] Gil RJ , Bil J , Kern A , et al. Angiographic restenosis in coronary bifurcations treatment with regular drug eluting stents and dedicated bifurcation drug‐eluting BiOSS stents: analysis based on randomized POLBOS i and POLBOS II studies. Cardiovasc Ther. 2020;2020:1‐8. 10.1155/2020/6760205 PMC7204374 32411301

[22] Toth GT , Achim A , Kafka M , et al. Bench test and in vivo evaluation of longitudinal stent deformation during proximal optimisation. EuroIntervention. 2022;18:83‐90. 34930716 10.4244/EIJ-D-21-00824 PMC9904376

[23] Stone GW , Sabik JF , Serruys PW , et al. Everolimus‐Eluting stents or bypass surgery for left main coronary artery disease. N Engl J Med. 2016;375:2223‐2235. 27797291 10.1056/NEJMoa1610227

[24] Mäkikallio T , Holm NR , Lindsay M , et al. Percutaneous coronary angioplasty versus coronary artery bypass grafting in treatment of unprotected left main stenosis (NOBLE): a prospective, randomised, open‐label, non‐inferiority trial. The Lancet. 2016;388:2743‐2752. 10.1016/S0140-6736(16)32052-9 27810312

[25] Hildick‐Smith D , Egred M , Banning A , et al. The European bifurcation club left main coronary stent study: a randomized comparison of stepwise provisional vs. systematic dual stenting strategies (EBC MAIN). Eur Heart J. 2021;42:3829‐3839. 34002215 10.1093/eurheartj/ehab283

[26] Fath‐Ordoubadi F , Spaepen E , El‐Omar M , et al. Outcomes in patients with acute and stable coronary syndromes; insights from the prospective NOBORI‐2 study. PLoS One. 2014;9:e88577. 24551120 10.1371/journal.pone.0088577 PMC3925145

[27] Chung SC , Gedeborg R , Nicholas O , et al. Acute myocardial infarction: a comparison of short‐term survival in national outcome registries in Sweden and the UK. The Lancet. 2014;383:1305‐1312. 10.1016/S0140-6736(13)62070-X PMC4255068 24461715

[28] Chan MY , Sun JL , Newby LK , et al. Long‐term mortality of patients undergoing cardiac catheterization for ST‐elevation and non‐ST‐elevation myocardial infarction. Circulation. 2009;119:3110‐3117. 19506116 10.1161/CIRCULATIONAHA.108.799981

[29] Heestermans AACM , van Werkum JW , Zwart B , et al. Acute and subacute stent thrombosis after primary percutaneous coronary intervention for ST‐segment elevation myocardial infarction: incidence, predictors and clinical outcome. J Thromb Haemostasis. 2010;8:2385‐2393. 20831622 10.1111/j.1538-7836.2010.04046.x

[30] Han Y , Liu H , Yang Y , et al. A randomised comparison of biodegradable polymer‐and permanent polymer‐coated platinum‐chromium everolimus eluting coronary stents in China: the EVOLVE China study. EuroIntervention. 2017;13:1210‐1217. 28741576 10.4244/EIJ-D-17-00271

[31] Ploumen EH , Pinxterhuis TH , Buiten RA , et al. Final 5‐year report of the randomized BIO‐RESORT trial comparing 3 contemporary drug‐eluting stents in all‐comers. J Am Heart Assoc. 2022;11:26041. 10.1161/JAHA.122.026041 PMC9750072 36346050

[32] Kereiakes DJ , Meredith IT , Windecker S , et al. Efficacy and safety of a novel bioabsorbable polymer‐coated, everolimus‐eluting coronary stent. Circul Cardiovasc Interv. 2015;8:e002372. 10.1161/CIRCINTERVENTIONS.114.002372 25855680

