# Supplementary material for: The everolimus eluting Synergy MegatronTM drug‐eluting stent platform: Early outcomes from the European Synergy MegatronTM Implanters' Registry
Source: Catheter Cardiovasc Interv. 2023 Nov 10;102(7):1222–8. doi: 10.1002/ccd.30902 (PMC10903108; doi:10.1002/ccd.30902)
Supplement: Supplementary file 1 — Supporting information. [file CCD-102-1222-s001.docx]

**Supplemental Table 1. Baseline Patient Characteristics Stratified by Target Lesion Complexity**

|  | **Complex (n=410)** | **Non-complex (n=165)** | **p-value** |
| --- | --- | --- | --- |
| Age (years) | 69.6±11.5 | 65.6±11.1 | <0.0001* |
| Male | 319 (80) | 137 (90) | 0.002* |
| Hypertension | 256 (64) | 85 (56) | 0.028 |
| Dyslipidaemia | 244 (61) | 84 (55) | 0.131 |
| Diabetes | 112 (28) | 30 (20) | 0.026* |
| End Stage Renal Failure | 16 (4) | 3 (2) | 0.208 |
| Previous MI | 112 (28) | 27 (18) | 0.019* |
| Previous PCI | 136 (34) | 38 (25) | 0.424 |
| Previous CABG | 36 (9) | 14 (9) | 0.872 |
| Previous CVA | 48 (12) | 9 (6) | 0.032* |
| PVD | 41 (10) | 14 (9) | 0.536 |
| BMI | 27.7±6.5 | 28.6±8.0 | 0.848 |
| LV function |  |  |  |
| Normal | 225 (57) | 88 (60) | 0.293 |
| Mild-Moderate impairment | 123 (30) | 50 (30) | 0.840 |
| Severe impairment | 46 (12) | 8 (5) | 0.028* |
| Clinical Syndrome |  |  |  |
| Stable angina | 169 (43) | 54 (35) | 0.450 |
| ACS - NSTEMI/UA | 167 (42) | 48 (31) | 0.003* |
| ACS - STEMI | 61 (15) | 51 (33) | <0.0001* |

Complex defined as a left main, ostial RCA or bifurcation target lesion. BMI: body mass index; CABG: coronary artery bypass graft; CVA: cerebrovascular accident; MI: myocardial infarction; NSTEMI: non-ST elevation myocardial infarction; PCI: percutaneous coronary intervention; PVD: peripheral vascular disease; ST elevation myocardial infarction.
